# The Semen pH Affects Sperm Motility and Capacitation

**DOI:** 10.1371/journal.pone.0132974

**Published:** 2015-07-14

**Authors:** Ji Zhou, Li Chen, Jie Li, Hongjun Li, Zhiwei Hong, Min Xie, Shengrong Chen, Bing Yao

**Affiliations:** 1 Center for Reproductive Medicine, Jinling Hospital, Clinical School of Medical College, Nanjing University, Nanjing, People's Republic of China; 2 Department of Neurosurgery, Jinling Hospital, Clinical School of Medical College, Nanjing University, Nanjing, People's Republic of China; 3 Department of Urology, Peking Union Medical College Hospital, Chinese Academy of Medical Sciences and Peking Union Medical College, Beijing, People's Republic of China; Clermont-Ferrand Univ., FRANCE

## Abstract

As the chemical environment of semen can have a profound effect on sperm quality, we examined the effect of pH on the motility, viability and capacitation of human sperm. The sperm in this study was collected from healthy males to avoid interference from other factors. The spermatozoa cultured in sperm nutrition solution at pH 5.2, 6.2, 7.2 and 8.2 were analyzed for sperm total motility, progressive motility (PR), hypo-osmotic swelling (HOS) rate, and sperm penetration. Our results showed that these parameters were similar in pH 7.2 and 8.2 sperm nutrition solutions, but decreased in pH 5.2 and 6.2 solutions. The HOS rate exhibited positive correlation with the sperm total motility and PR. In addition, the sperm Na^+^/K^+^-ATPase activity at different pHs was measured, and the enzyme activity was significantly lower in pH 5.2 and 6.2 media, comparing with that in pH 8.2 and pH 7.2 solutions. Using flow cytometry (FCM) and laser confocal scanning microscopy (LCSM) analysis, the intracellular Ca^2+^ concentrations of sperm cultured in sperm capacitation solution at pH 5.2, 6.2, 7.2 and 8.2 were determined. Compared with that at pH 7.2, the mean fluorescence intensity of sperm in pH 5.2 and 6.2 media decreased significantly, while that of pH 8.2 group showed no difference. Our results suggested that the declined Na^+^/K^+^-ATPase activity at acidic pHs result in decreased sperm movement and capacitation, which could be one of the mechanisms of male infertility.

## Introduction

According to reported statistics, approximately one in six couples is infertile, of which nearly half attribute to male factors [1]. The main clinical manifestations of male infertility include reduced sperm count (oligozoospermia), decreased sperm motility (asthenospermia), and sperm morphological abnormalities (teratozoospermia) [2,3]. Over 85% of infertile males can actually produce sperm [4], but their sperms are often unable to fertilize an egg.

Sperm is the only human cell which performs its function outside the male body. The microenvironment of sperm, seminal plasma, is of great significance. Seminal plasma is a mixture of secretions from the testes, epididymides and accessory sex glands. Seminal plasma contains HCO_3_^-^/CO_2_, inorganic ions, organic acids, sugars, lipids, steroids, amino acids, polyamines, nitrogenous bases and proteins [5]. As a result, semen has a very high buffering capacity, higher than that of most other body fluids. Therefore, the pH of the seminal fluid may play a significant role not only in maintaining the viability and quality of the sperm, but also in ensuring fertilization. The effects of pH on sperm movement in birds, fish and shellfish have been investigated. For instance, the percentage of motile sperm and sperm velocity were increased at alkaline pH in Turkey and quail [6]. Similar results were obtained in domestic chickens (*Gallus gallus*), and alkalinization of the external pH restored sperm motility at 40°C [7,8]. Huang et al. (2001) reported that the sperm of *Anodonta woodiana Pacifica Houde* exhibited stronger motility at pH 8.5, which significantly declined at pH 7.0, 7.5, 9.5, and 10.0 [9]. Liu et al. (2010) reported that the sperm of *Crossochetlius hemispinus* has a weak adaptive capacity to different pH values which can move appropriately only in pH 6.5–7.5 solution [10]. However, the studies of human semen pH often focus on clinical cases. It has been reported that semen pH is lower than 7.2 in patients with oligospermia or/and asthenospermia [11]. Another study which divided its cohort into normal spermatozoa motility and hypomotility male observed no significant difference in seminal plasma pH between the two groups [12]. Based on these reports, we used sperm of healthy donors and explored the effect of different pH on the motility and viability of human sperm.

The Na^+^/K^+^-ATPase, also called Na pump, is an enzyme on plasma membrane that is responsible for the exchange of three cytoplasmic Na^+^ for two extracellular K^+^ using energy from the hydrolysis of ATP [13,14]. The Na^+^/K^+^-ATPase is consisted of two major polypeptides, α and β subunits [15]. The α subunit has four isoforms α1–4, and the α1 and 4 hav1. Please note that the Symbol font on pages 4, 7, 8, 9, and 13 cannot be typeset and will not render correctly in the published manuscript. Instead, you must use the “Insert>Symbol” function for another font type. Please remove all Symbol font from your manuscript. You can identify instances of Symbol font by using the “Find” function in Word and searching for “Format>Font>Symbol.” To correctly replace the symbol, go to "Insert>Symbols", choose a text-based font such as "Times New Roman" or "Normal", and choose the appropriate symbol, ensuring that the font (typeface) does not remain Symbol.e been identified in testis and spermatozoa [16,17]. The α4isoform maintains the motility of male gametes primarily by controlling the transmembrane Na^+^ gradient in sperm, and that ouabain inhibition of α4caused motility decline of the sperm. Moreover, the α4isoform can functionally couple with the Na^+^/H^+^ exchanger (NHE) to regulate intracellular pH in spermatozoa, which affects the functions of both invertebrate and vertebrate sperm, such as motility or the initiation of the acrosome reaction [18–20]. A demonstrable increase in internal pH was shown to stimulate boar [21] and chicken [22] sperm motility. In this study, we try to explore the effects of changing pH of semen, whether it causes the change of sperm motility and intracellular pH, and whether this change is related to the Na pump function. In addition, the Na^+^/K^+^-ATPase activity is also necessary for maintaining the intracellular Ca^2+^ concentration [23]. The major parameters of sperm function, such as the flagella swing, swimming speed, sperm capacitation, and acrosome reaction, are affected by Ca^2+^ influx, and the sperm motility depends on the intracellular free Ca^2+^ concentration, which leads to sperm superactivation. Munire *et al*. [24] reported that weakened sperm movement and superactivation may be associated with infertility; the amplitude of lateral head displacement (ALH) in human sperm is positively correlated with the increase in Ca^2+^ influx.

Some clinical case reports indicate that the semen pH of asthenospermia male may have changed [11,25]. However, whether and how the semen pH affects sperm movement and capacitation remains unclear. Understanding the effects of seminal plasma pH may be helpful for the clinical treatment of infertility. Therefore, we investigated the changes in sperm motility and viability over a biologically relevant pH range, and measured the corresponding Na^+^/K^+^-ATPase activity and intracellular Ca^2+^ changes of sperms.

## Materials and Methods

### Sperm collection

This study was approved by the Ethics Committee of Nanjing Jinling Hospital and in accordance with National and International guidelines. A total of 136 male volunteers participated in this study, with a mean age of 27.54 ± 3.98 (mean ± standard deviation). Before initiating the study, written informed consent was obtained from all participants. The sperm subjected to analysis were from healthy male volunteers. These men had proven fertility and normal semen quality, according to the World Health Organization criteria (2010). The semen samples were obtained by masturbation after at least 3 days of abstinence.

The samples were ejaculated into sterile containers and allowed to liquefy for at least 30 min before being processed by centrifugation in a 60% Percoll gradient (GE Healthcare, Waukesha, WI, USA) to remove seminal plasma, immature germ cells, and non-sperm cells (mainly epithelial cells), as described by Loredana-Gandini et al. [26,27]. The purified sperms were then washed in sperm nutrition solution (NaCl 670.8 mg; KCl 35.6 mg; 147 mg/mL CaCl_2_·2H_2_O 171 μL; KH_2_PO_4_ 16.3mg; MgSO_4_·7H_2_O 29.3 mg; NaHCO_4_ 210.6 mg; D-glucose 100 mg; 60% Na·lactate 0.37 mL; HEPES 238 mg; Penicllin 6 mg, dissolved in 100 mL sterile water) 3 times before subsequent analysis. All of the following experiments used purified sperm pooled from 3–4 volunteers assigned to four groups, and all experiments were performed at least in three replicates.

### Assessment of sperm motility

Purified sperms were resuspended in sperm nutrition solution of pH 5.2, 6.2, 7.2 and 8.2, and incubated for 15, 30, 60, 90 and 120 min, respectively. Computer-assisted semen analysis (CASA; WLJY-9000; Weili New Century Technology Development Co., Ltd.; Beijing; China) was used to assess the sperm motility. Non-progressive motility (NP, all other patterns of motility with an absence of progression), progressive motility (PR, spermatozoa moving actively, either linearly or in a large circle, regardless of speed), motility (PR + NP), average path velocity (VAP, velocity over a calculated smoothed path), curvilinear velocity (VCL, velocity over the actual sperm track, including all deviations of sperm head movement), straight line velocity (VSL, velocity over the straight line distance between the beginning and the end of the sperm track) were calculated for each group from the three recordings of at least 100 sperm.

### HOS test

The HOS test was used in sperm viability assessment. Sperm were resuspended in sperm nutrition solution of pH 5.2, 6.2, 7.2 and 8.2, respectively, for 60 min, and centrifuged at 760 g for 15 min. The supernatant fluid was discarded, and the precipitate was mixed with 0.5 mL swelling solution (0.735 g sodium citrate dihydrate and 1.351 g D-fructose dissolved in 100 mL purified water) followed by incubation for 30 min at 37°C. After incubation, more than 200 spermatozoa were examined by CASA. According to the World Health Organization criteria (2010), the number of sperm swollen and un-swollen tails were recorded respectively [28].

### Sperm penetration meter (SPM) test

Capillaries full of sperm nutrition solution with different pH were perpendicularly inserted into test tubes containing 200 μL spermatic fluid (purified human sperm in sperm nutrition solution). The top of capillary pipettes and the opening of test tubes were all sealed up with seal film followed by incubation for 1 h at 37°C. According to the World Health Organization criteria (2010), the movement distance of sperm was measured under optical microscope (CX31; OLYMPUS; Japan).

### Na^+^/K^+^-ATPase assay

Sperm (10^7^) were diluted with 2 mL sperm nutrition solutions at pH 5.2, 6.2, 7.2 and 8.2, respectively, mixed vigorously, and incubated for 60 min at 37°C in a 5% CO_2_ incubator. The tubes were centrifuged at 760 g for 15 min to remove the nutrition solution, and the precipitation was washed with normal saline solution for 3 times and lysed by ultrasonication (4°C,40% of the energy, sonicated for 5 s, stop for 5 s, a cycle of 1 min) (VCX750; Sonics; USA). The protein concentration was determined by bicinchoninic acid (BCA) assay (product No. 23225, Thermo, USA). The Na^+^/K^+^-ATPase activity of the sperm was measured by Na^+^/K^+^-ATPase Measurement Kit (Nanjing Jiancheng Bioengineering Inc.), and the unit was demonstrated as μmolPi/mg prot/hour.

### Intracellular Ca^2+^ assay

The level of intracellular Ca^2+^ of sperm were determined by flow cytometry (FCM) and laser confocal scanning microscopy (LSCM).

Sperm were incubated in capacitating media (sperm nutrition solutions with 10 mg/mL bovine serum albumin [BSA; No.A8806; Sigma; USA]) [29], at pH 5.2, 6.2, 7.2, and 8.2, respectively. After incubation for 3.5 h at 37°C in a 5% CO_2_ incubator, 200 μL supernatant containing the capacitated sperm were taken and adjusted to the sperm concentration of 2×10^6^/mL. These samples were prepared for sperm intracellular Ca^2+^ assay.

Briefly, 200 μL sperm in capacitation solution was centrifuged at 3,000 rpm for 5 min; the supernatant was discarded, and the sperm were resuspended in 300 μL 0.01 mol/L PBS (Ca^2+^ and Mg^2+^ plus). The samples were then incubated with 2 μM Fluo3-AM (Molecular Probe Inc. USA) for 30 min at 37°C, washed with PBS, and finally resuspended in 1 mL PBS with Ca^2+^ and Mg^2+^.

The suspended semen sample stained with Fluo3-AM (0.8 mL) was analyzed by FCM on a FACS Calibur (Becton Dickinson, USA). A total of 20,000 cells were measured for each sample, and the fluorescence intensity of every individual spermatozoon was obtained. The changes of intracellular Ca^2+^ were expressed as average fluorescence divided by background fluorescence. The obtained data were finally analyzed by Cell Quest software (Version 3.2.1, Becton Dickinson Immunocytometry Systems, Silicon Valley, CA).

LSCM (ZEISS LSCM510, Germany) was also used to measure the relative fluorescence intensity in individual spermoplasm added with Fluo3-AM. The fluorescence was recorded at excitation wavelength of 488 nm and emission wavelength of 530 nm. Time series of optical sections of a cell was obtained with a XY-step. The fluorescence measurements for different fields of view were performed under 400× objective, and the mean fluorescence intensity of all sperm cells in one field of view was used to represent the fluorescence intensity of this semen sample.

### Statistical analysis

SPSS 13.0 software (SPSS Inc., Chicago IL, USA) was used for statistical evaluation. Linear regression was used to estimate the relationship between the movement of sperm and HOS rate. Other results (motility, PR, HOS rate, SPM, Na^+^/K^+^-ATPase and Intracellular Ca^2+^ concentrations) were compared using one-way ANOVA method, and the homogeneity of variance was tested before (with 0.1 as the standard test conducted Levene test). When equal variances were assumed, the LSD test was selected. Otherwise, a Dunnett’s T3 test was performed. The data were expressed as means ± standard deviation. The differences were considered statistically significant when p < 0.05.

## Results

### Sperm movement assessment after treatment with nutrition solutions at different pHs

**Fig 1:**
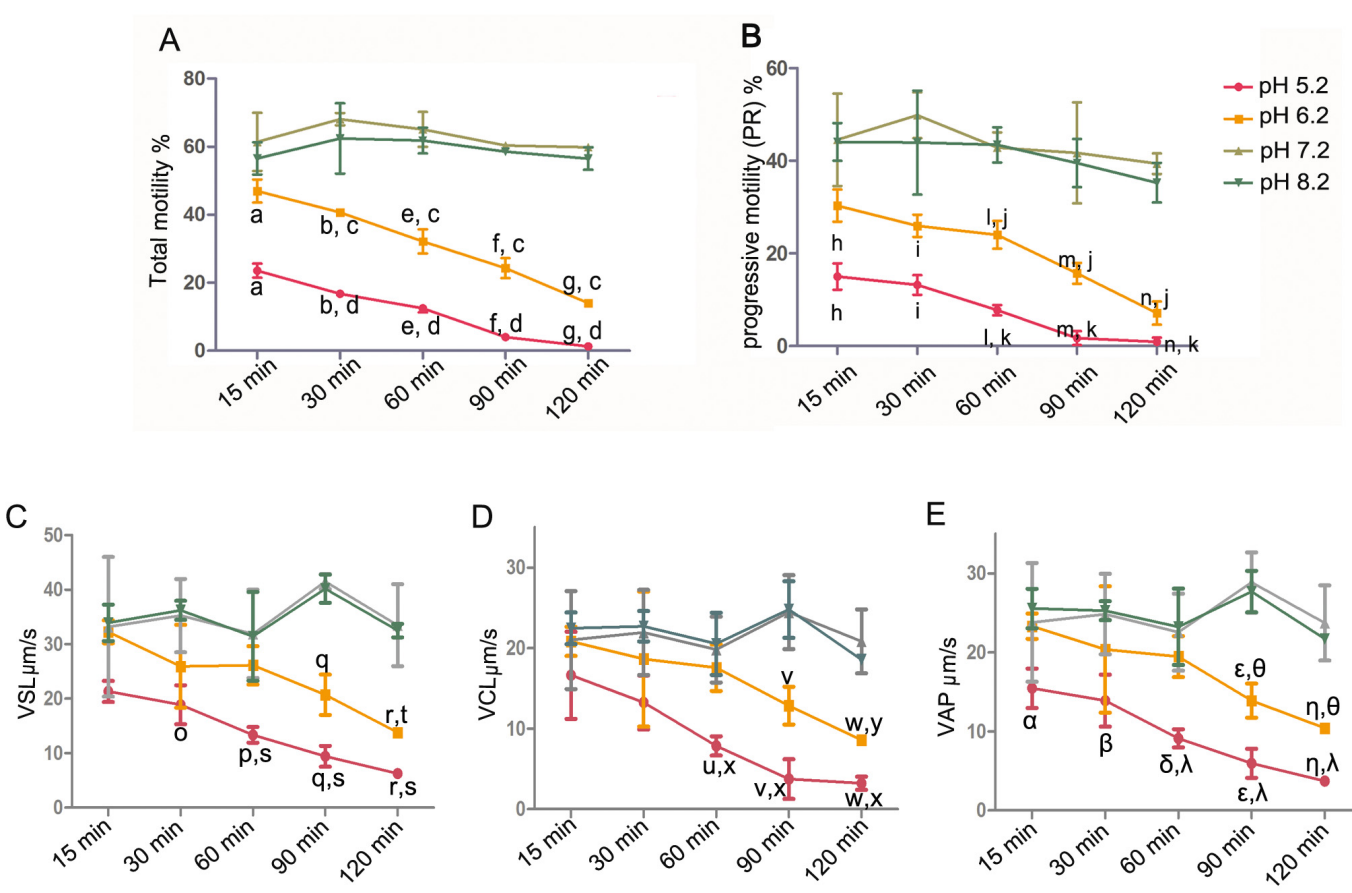
Effect of pH on the motility and velocity of sperm. Purified human sperm were incubated in pH 5.2, 6.2, 7.2 and 8.2 nutrient solutions for 15, 30, 60, 90 and 120 min, respectively. (A) Sperm mobility (PR + NP): ^a,b,e-g^ p<0.05, statistically significant different from pH 7.2 group at 15, 30, 60, 90, and 120 min, respectively; ^c^ p<0.05, statistically significant different from pH 6.2 group at 15 min; ^d^p<0.05, statistically significant different from pH 5.2 group at 15 min. (B) PR: ^h,i,l-n^ p<0.05, statistically significant different from pH 7.2 group at 15, 30, 60, 90, and 120 min, respectively; ^j^p<0.05, statistically significant different from pH 6.2 group at 15 min; ^k^p<0.05, statistically significant different from pH 5.2 at 15 min. (C) mean straight line velocity (VSL): ^o-r^p<0.05, statistically significant different from pH 7.2 group at 30, 60, 90 and 120 min, respectively; ^t^ p<0.05, statistically significant different from pH 6.2 group at 15 min; ^s^p<0.05, statistically significant different from pH 5.2 group at 15 min. (D) mean curvilinear velocity (VCL): ^u-w^ p<0.05, statistically significant different from pH 7.2 group at 60, 90 and 120 min, respectively; ^y^ p<0.05, statistically significant different from pH 6.2 group at 15 min; ^x^p<0.05, statistically significant different from pH 5.2 group at 15 min. and; (E) mean average path velocity (VAP): ^α, β, δ, ε, η^ p<0.05, statistically significant different from pH 7.2 group at 15, 30, 60, 90 and 120 min, respectively; ^θ^ p<0.05, statistically significant different from pH 6.2 group at 15 min; ^λ^p<0.05, statistically significant different from pH 5.2 group at 15 min (N = 3).

As the human semen pH is in the range from 5.2 to 8.2 [30], purified human sperm were incubated in pH 5.2, 6.2, 7.2 and 8.2 nutrient solutions for 15, 30, 60, 90 and 120 min, respectively, in order to evaluate the effect of pH on sperm movement. As shown in Fig 1A, the total motility of sperm decreased only slightly during the entire incubation period of 120 min (pH 7.2: 59.86 ± 0.85%; pH 8.2: 56.52 ± 3.30%) in pH 7.2 and 8.2 nutrient solutions. In contrast, the acidic environment (pH 5.2 and 6.2) significantly reduced the percentage of total motile spermatozoa in a time-dependent manner. Moreover, the acidic environment (pH 5.2 and 6.2) also decreased PR of sperm (Fig 1B). Velocity parameters (VSL, VCL and VAP) were also assessed (Fig 1C, 1D and 1E). The velocity parameters were similar to the PR of sperm, i.e., the acidic environment (pH 5.2 and 6.2) decreased the VSL, VCL and VAP of sperm.

### The viability of sperm was decreased in acidic environments

**Fig 2:**
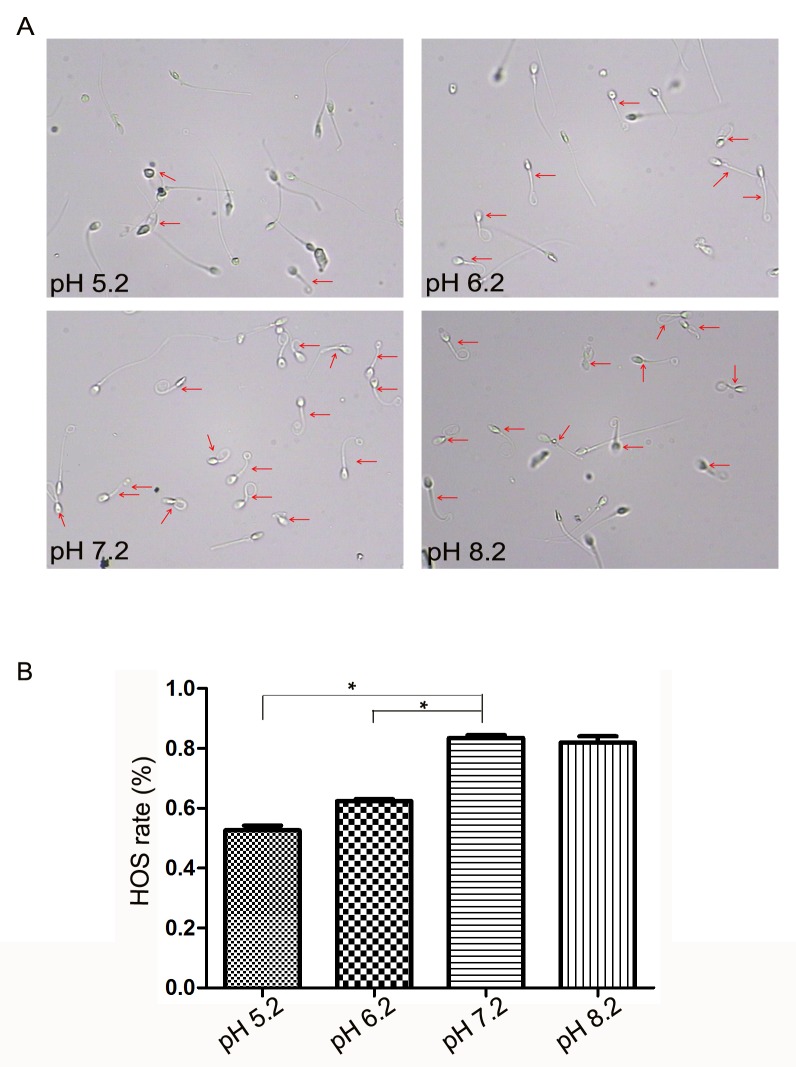
The viability of sperm in nutrition solutions with different pH. (A), the images of HOS spermatozoa in different pH nutrition solutions were obtained by CASA. (B), HOS rate markedly declined in pH 5.2 and 6.2 nutrition solutions in comparison with pH 7.2 solution, * mean p<0.01.

**Fig 3:**
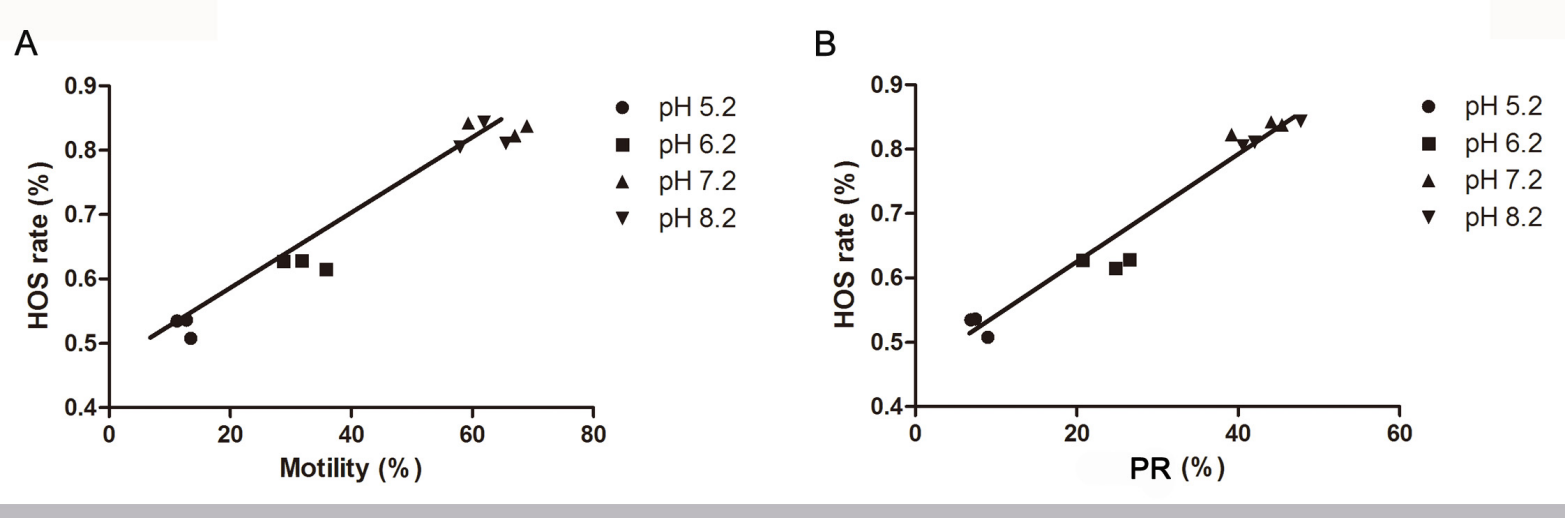
Linear regression between HOS rate and sperm movement. (A), the correlation between HOS rate and sperm mobility, r = 0.98, p<0.05. (B), the correlation between HOS rate and sperm PR, r = 0.98, p<0.05.

To explore whether the viability of sperm changed during the incubation in different pH nutrient solutions for 60 min, we determined the HOS rate of sperm, and the results showed that the HOS rate significantly declined in pH 5.2 (52.62 ± 0.02%, p<0.05) and 6.2 (62.35 ± 0.01%, p<0.05) nutrition solutions compared with pH 7.2 (83.43 ± 0.01%), but did not change in pH 8.2 (82.00 ± 0.02%, Fig 2B). Furthermore, the relationship between HOS rate and the movement of sperm in different pH was analyzed at 60 min. A positive correlation was found for the sperm total motility (r = 0.98, p<0.05, Fig 3A). The same was true for the correlation between HOS rate and the sperm PR (r = 0.98, p<0.05, Fig 3B).

### Sperm penetration decreased in acidic environments

**Fig 4:**
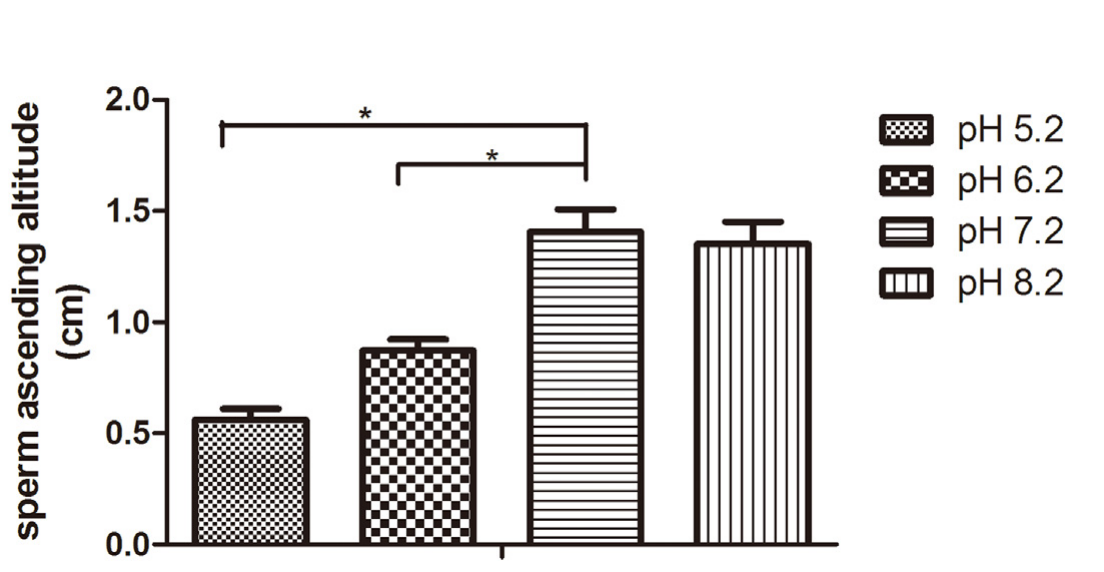
Sperm penetration decreased in acidic environments. Compared with pH 7.2 group, the sperm ascending altitude in pH 5.2 and 6.2 groups was significantly lower * p<0.01 (N = 9).

We found that sperm ascending altitude in pH 7.2 nutrition solution was higher than any other pH groups. Compared with pH 7.2 group (1.41 ± 0.10 cm), the sperm ascending altitude in pH 5.2 group (0.56 ± 0.05 cm) and in pH 6.2 (0.87 ± 0.05 cm) was significantly lower (p<0.01) (Fig 4).

### Sperm Na^+^/K^+^-ATPase activity decreased in acidic environments

**Fig 5:**
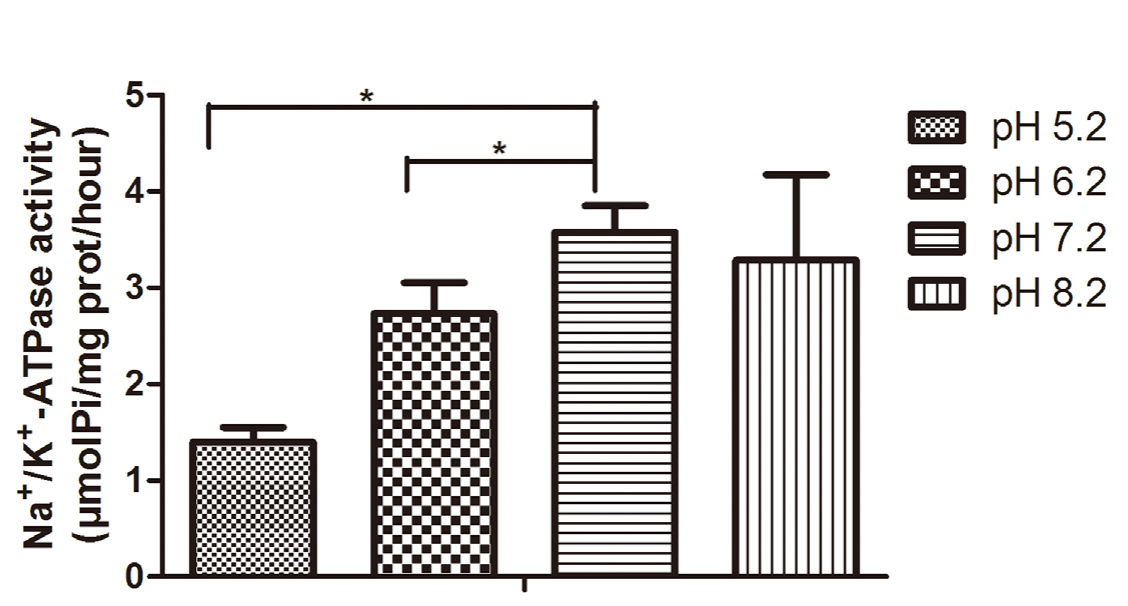
Sperm Na^+^/K^+^-ATPase activity decreased in acidic environments. Compared with pH 7.2, sperm Na^+^/K^+^-ATPase activity decreased evidently at pH 5.2 or 6.2, while there were no significant differences with that at pH 8.2. * p<0.05 (N = 4).

Compared with pH 7.2 (3.58 ± 0.28 μmolPi/mg prot/hour), sperm Na^+^/K^+^-ATPase activity decreased evidently under pH 5.2 (1.40 ± 0.15 μmolPi/mg prot/hour, p<0.05) or 6.2 (2.73 ± 0.32 μmolPi/mg prot/hour, p<0.05), while there was no significant changes with pH 8.2 (3.29 ± 0.89 μmolPi/mg prot/hour, Fig 5).

### Acidic environments reduced [Ca^2+^]_i_ during human sperm capacitation

**Fig 6:**
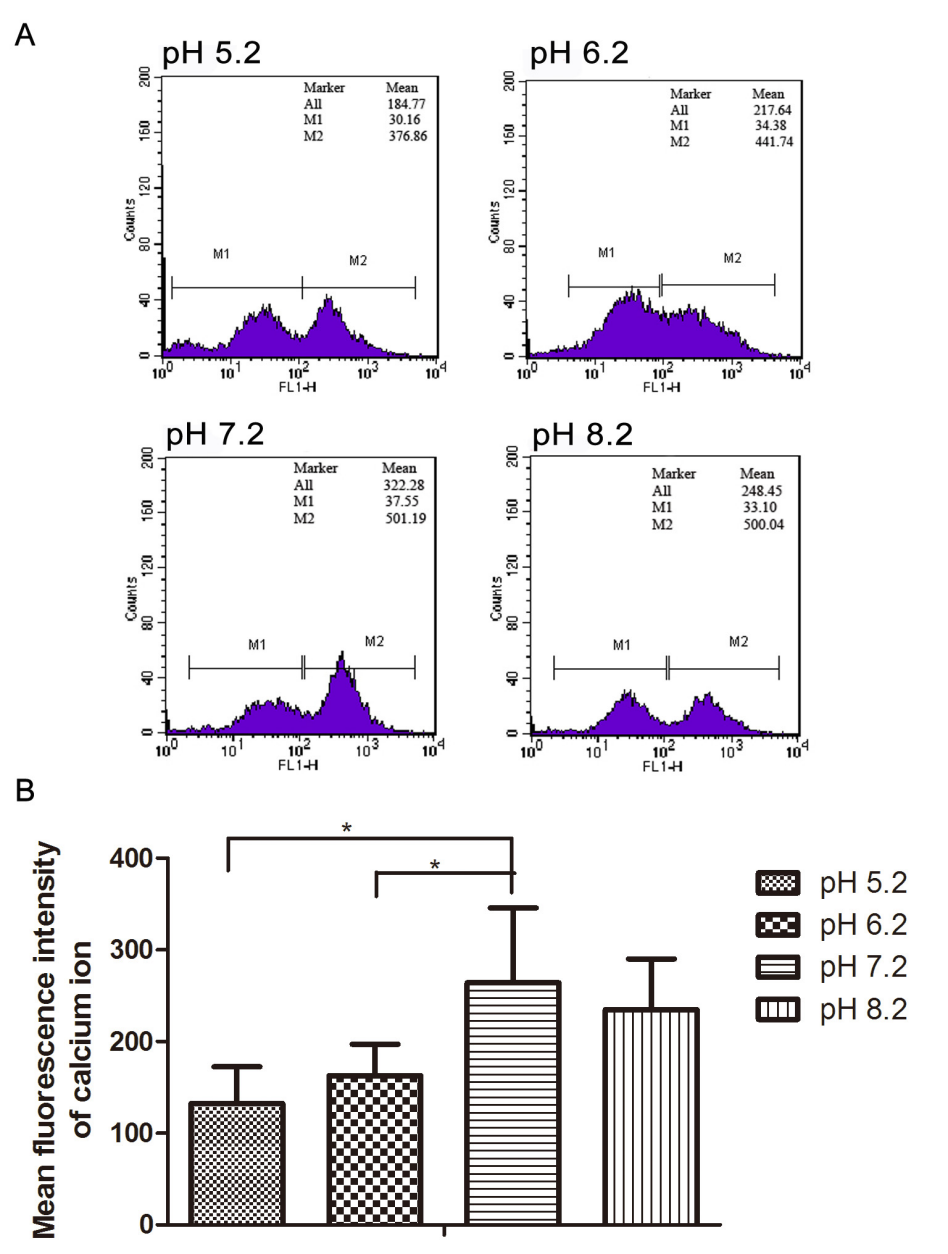
Measurement of intracellular Ca^2+^ during human sperm capacitation by FCM. (A), the FCM results of spermatozoa in sperm nutrition solutions at pH 5.2, 6.2, 7.2 and 8.2, respectively. (B), compared with pH 7.2 group, the mean fluorescent intensity of intracellular Ca^2+^ in pH 5.2 and 6.2 groups significantly declined, but the pH 8.2 group did not show significant differences. * p<0.05 (N = 6)

Fig 6 shows the intracellular Ca^2+^ level of sperms in sperm capacitation solutions at different pH were analyzed by FCM. The fluorescence intensity of intracellular Ca^2+^ (264.15 ± 81.57) was peaked at pH 7.2. Compared with pH 7.2, the fluorescence intensities of intracellular Ca^2+^ in other groups (132.55 ± 40.11 at pH 5.2; 162.76 ± 34.34 at pH 6.2) significantly declined except for pH 8.2 (234.82 ± 55.24).

**Fig 7:**
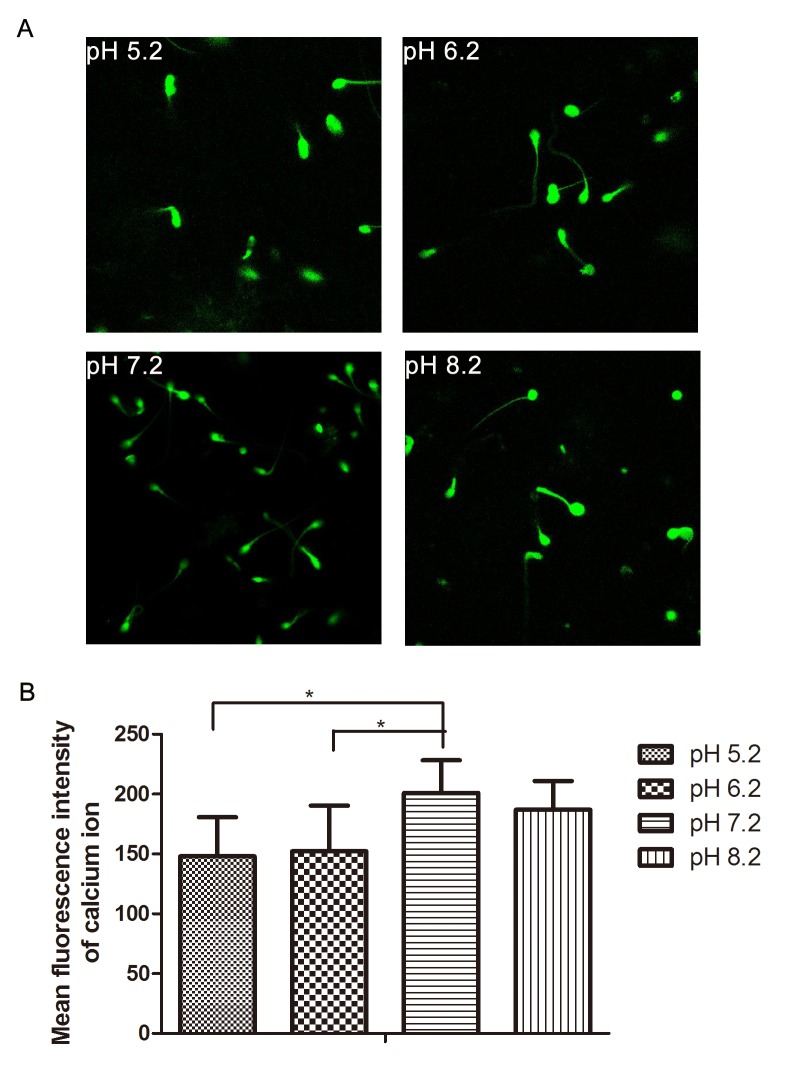
Measurement of intracellular Ca^2+^ during human sperm capacitation by LSCM. (A), the LSCM results of spermatozoa in sperm nutrition solutions at pH 5.2, 6.2, 7.2 and 8.2, respectively. (B), compared with pH 7.2 group, the mean fluorescent intensity of intracellular Ca^2+^ in pH 5.2 and 6.2 groups significantly declined, but the pH 8.2 group did not show significant differences. * p<0.05 (N = 10)

LSCM observation also confirmed the data obtained by FCM; the results of LSCM showed that the fluorescence intensity of intracellular Ca^2+^ reached the maximum at pH 7.2 (200.87 ± 27.43; Fig 7). Weak fluorescent signals were observed in sperm cytoplasm at both pH 5.2 (147.93 ± 32.90) and 6.2 (152.18 ± 38.05), suggesting that acidic environment lead to a significantly lower concentration of intracellular Ca^2+^, and affected sperm capacitation.

## Discussion

Sperm motility is a critical factor in determining semen quality and fertilizing ability. And the chemical environment of semen can have a profound effect on sperm quality. However, it has been shown that over 85% of infertile males can actually produce sperms that have abnormal functions, suggesting that except for sperm itself, additional factors such as seminal plasma pH, inorganic phosphate, total and ionized calcium concentrations, and others could contribute to male fertility. In this study, we used purified sperm from healthy male volunteers to avoid the interference from other factors in seminal plasma and the presence of abnormal sperm. And the effect of pH on sperm quality and investigated the possible mechanism were explored.

This study revealed that pH 7.2 and 8.2 appeared to be the optimal condition for total motility and PR of human sperm. In addition, velocity parameters (VSL, VAP and VCL) significantly decreased at pH 6.2 and 5.2, which was consistent with the result of motility. Additionally, fertilization success was always found to be correlated with VSL, VAP and VCL in the chicken, turkeys and human [31–33]. Our study showed that in acidic environments (pH 5.2 and 6.2), most of the sperm, which stayed within 1 cm from basal part of capillary pipettes, swam *in situ*, and few of them swam upward in the sperm penetration meter test. These results suggested that pH of semen can directly affect sperm movement. In a clinical research, with a sample size of 360 infertile men, significantly reduced sperm concentration, motility, and PR (lower than the WHO standard) were observed in the decreased semen pH (p<0.001); additionally, increased abnormal sperm rate existed in the declined semen pH, too (p<0.001) [25]. Qi *et al*. tested pH of semen in 111 infertile cases and found that 71 patients (64.0%) were with semen pH < 6.8 [34]. In fish and avian species, pH has also been reported as one of the major sperm fertilizing factors [35]. In Scaphthalmus maximus, pH 9.0 was considered suitable for the maximum sperm motility [36]. The percentage of motile sperm in Petromyzon marinus did not change over the pH range 6.0–9.0 [37]. The motility of spermatoza from chicken, turkey and quail was inhibited at pH below 7.8, 7.2 and 7.2, respectively [6]. These reports are consistent with our results, confirming that the low pH of semen is related with decreased sperm movement and male infertility. However, another clinical research found that there was no significant difference in the pH of seminal plasma between the hypomotile and normal motility groups (p>0.05) [12]. This report did not agree with our findings, possibly because there was only 39 males in the hypomotility group, and the other factors such as inorganic phosphate and ionized calcium concentrations could be the main factor in determining sperm fertilization. Our study, with the removal of other factors, might better illustrate the effect of pH on human sperm motility.

Since pH affects the metabolic rate and the motility of sperm, and consequently alters the vitality of sperm [38], we used the HOS test (the WHO recommended) to determine sperm vitality. We found that higher proportions of live normal sperm were observed in a neutral and alkaline (pH 7.2 and 8.2) environment. Lin *et al*. demonstrated that the HOS test was significantly positively correlated with sperm motility [39]. Consistent with this research, our data also showed a significant positive correlation between HOS and sperm movement, suggesting that abnormal pH (pH 6.2 and 5.2) shorten the lifespan of sperme and impair cell membrane. However, there had been different data on the pH and vitality, especially in birds. In ostrich, sperm mixed with diluents at pH 6.0 showed lower sperm motility and higher viability in comparison with pH 9.0 [38]. It is thought that the reduction in sperm metabolism conserves energetic resources promoting lifespan [38]. The findings of this report is different from ours, possibly because the sperm of ostrich was only incubated 10 min in pH 6.0 diluents, and pH 6.0 was within the scope of pH the ostrich sperm can tolerate. Our data suggested that different from ostrich sperm, human sperm can tolerate a very narrow range of pH, and weak acid (pH 6.2) was able to decrease the vitality significantly.

Na^+^/K^+^-ATPase is found on most mammalian cell membrane [40], and many basic and specialized cell functions have been attributed to Na^+^/K^+^-ATPase, which transfers hydrolysis energy of ATP to sustain the membrane potential [41]. Further characterization shows that the distribution of Na^+^/K^+^-ATPase in the spermatozoa is restricted to the flagella membrane located at the middle and posterior part of the spermatozoa [42]. The causes for infertility may arise from reduced Na^+^/K^+^-ATPase activity of sperm plasma membrane. In asthenospermic males, compared with normal sperm, Na^+^/K^+^-ATPase activity has been found significantly decreased [43]. Our study found that Na^+^/K^+^-ATPase activity in sperm membrane notably decreased in pH 5.2 and 6.2 sperm nutrition solution, suggesting that low pH affect sperm motility possibly via downregulating Na^+^/K^+^-ATPase activity. Besides its direct role in Na^+^ and K^+^ transport, the Na^+^/K^+^-ATPase secondarily controls proton levels in spermatozoa [23]. The Na^+^/K^+^-ATPase can functionally couple with the Na^+^/H^+^ exchanger (NHE) to regulate pH in sperm [23]. It has been reported that intracellular pH is a critical regulator of sperm motility [43]. When NH_4_Cl [44] or bicarbonate [45] alone was added to the external medium, both of which stimulate H^+^ release and are sufficient to induce sperm motility. Using ouabain to specifically inhibit the function of the Na^+^/K^+^-ATPase induces intracellular acidification of sperm by eliminating Na^+^ gradients necessary for the Na^+^/H^+^ exchanger to remove excess H^+^, resulting in the loss of motility [16,23]. Our study suggested that the acidic environment (pH 5.2 and 6.2) decrease Na^+^/K^+^-ATPase activity, maybe similar to ouabain, result in a more acidic cytoplasm in immobile sperm, which requires futher research to confirm.

Calcium is important for sperm motility, metabolism, acrosome reaction, hyperactivated motility and fertilization [46]. Changes in intracellular Ca^2+^ concentration are associated with different aspects of sperm function [12]. Massive Ca^2+^ influx and Ca^2+^ induces hyperactivated motility of sperm during acrosome reaction [47,48]. When hyperactivated sperm are transferred to Ca^2+^-free medium for 30–60 min, hyperactivated movement stops, but addition of 2 mM Ca^2+^ restores the hyperactivated motility [49]. Ca^2+^ can enhance the sperm's flagellar beat amplitude, and there is a direct relationship between the intracellular Ca^2+^ level and hyperactivated movement [50,51]. It was reported that Na^+^/K^+^-ATPase was able to participate in intracellular calcium homeostasis [23,52], and its activity increased during capacitation [53]. Our results showed that the low Na^+^/K^+^-ATPase activity in pH 5.2 and 6.2 medium would impair sperm capacitation. After measuring the level of intracellular Ca^2+^, we found that the level of intracellular Ca^2+^ was dramatically reduced under pH 5.2 and 6.2 compared to pH 7.2, which may result in declined Na^+^/K^+^-ATPase activity, and then decreased Ca^2+^ influx. Under a low Ca^2+^ condition, the acrosome reaction cannot be initiated, ultimately affecting fertilization.

Our results showed that the pH of semen affected sperm motility, implying that the pH of vaginal microenvironment in women may also affect sperm activity. After ejaculation, sperm pass the female reproductive tract with seminal plasma; therefore spermatozoon is vulnerable to various chemicals in both semen and female reproductive tract, and these chemicals may directly affect sperm motility and metabolism, and then influence the whole process of capacitation and fertilization as well [54]. The alteration of pH not only occurs in male semen but also in female vagina under infections, such as trichomoniasis, bacterial vaginosis, and cytolytic vaginosis [55], which may affect sperm motility and capacitation, and even result in infertility.

## Conclusions

In conclusion, we found slightly alkaline conditions seem to stimulate human sperm motility and capacitation. The dramatically decreased Na^+^/K^+^-ATPase activity caused by acidic environment could be the reason of lower sperm motility and Ca^2+^ level, which ultimately affected pregnancy. However, the mechanisms of pH affecting Na^+^/K^+^-ATPase activity in human spermatozoa are not completely elucidated. In addition to affecting Na^+^/K^+^-ATPase, acidic environment may be able to damage the sperm cell membrane directly, or to increase the active oxygen content, thus affecting sperm motility and capacitation, which warrant further explorations.
